# Temozolomide-Induced Shrinkage of Invasive Pituitary Adenoma in Patient with Nelson's Syndrome: A Case Report and Review of the Literature

**DOI:** 10.1155/2015/623092

**Published:** 2015-06-29

**Authors:** Maria Kurowska, Andrzej Nowakowski, Grzegorz Zieliński, Joanna Malicka, Jerzy S. Tarach, Maria Maksymowicz, Piotr Denew

**Affiliations:** ^1^Department of Endocrinology, Medical University of Lublin, Lublin, Poland; ^2^Department of Neurosurgery, Military Medical Institute, Warsaw, Poland; ^3^Department of Pathology, M. Sklodowska-Curie Memorial Cancer Centre and Institute of Oncology, Warsaw, Poland

## Abstract

*Introduction*. Invasive tumours in Nelson's syndrome need aggressive therapy. Recent reports have documented the efficacy of temozolomide (TMZ) in the treatment of adenomas resistant to conventional management. *Objective*. The review of the literature concerning TMZ treatment of atypical corticotroph adenomas and a case study of 56-year-old woman who developed Nelson's syndrome. *Treatment Proceeding*. The patient with Cushing's disease underwent transsphenoidal adenomectomy followed by a 27-month-long period of remission. Due to a regrowth of the tumor, she underwent two reoperations followed by stereotactic radiotherapy. Because of treatment failures, bilateral adrenalectomy was performed. Then she developed Nelson's syndrome. A fourth transsphenoidal adenomectomy was performed, but there was a rapid recurrence. Five months later, she underwent a right frontotemporal craniotomy. Due to a rapid regrowth of the tumour, the patient did not receive gamma-knife therapy and was treated with cabergoline and somatostatin analogue for some time. Only TMZ therapy resulted in marked clinical, biochemical, and radiological improvement. To date, this is the first case of invasive corticotroph adenoma in Nelson's syndrome treated with temozolomide in Poland. *Conclusion*. In our opinion, temozolomide can be an effective treatment option of invasive adenomas in Nelson's syndrome.

## 1. Introduction

Nelson's syndrome (NS) is a potentially life-threatening complication of bilateral adrenalectomy performed in patients with refractory Cushing's disease in the course of aggressive subtypes of corticotroph pituitary adenomas. The risk of developing NS in adults after bilateral adrenalectomy ranges from 8 to 47%. The main pathological feature of NS is locally invasive pituitary macroadenoma invading and compressing surrounding structures [1, 2].

Invasive corticotroph tumors in NS are associated with poor prognosis and their treatment remains a challenge. They require aggressive management and usage of many treatment methods because some of them behave like carcinomas. The treatment of choice is neurosurgical removal of the pituitary adenoma. In the event of failure after initial or repeated pituitary surgery, second-line treatments of regrowing adenomas include adjuvant radiotherapy, stereotactic radiosurgery, selective somatostatin analogues (pasireotide), dopamine agonists (cabergoline) or sodium valproate [1–3], and, finally, EGFR kinase tyrosine inhibitors (gefitinib) [3, 4] and an antiangiogenic agent, bevacizumab, a monoclonal antibody that inhibits vascular endothelial growth factor VEGF [5].

So far, medical therapies of Nelson's syndrome have generally been ineffective. Recent reports have documented the efficacy of temozolomide, an orally administered alkylating agent, against aggressive pituitary tumors resistant to conventional therapy [2, 3, 5–11]. The first successful using of temozolomide in treating pituitary carcinoma was reported in 2004 (according to Syro et al. [10]) and the first patients with pituitary adenomas treated with TMZ were described in 2006 [12, 13]. A case of using TMZ in a patient with NS was first reported in 2008 [14]. To the best of our knowledge, up to this day, the implementation of TMZ in NS has been reported in six patients only [10, 11, 14–17].

Temozolomide is a new second-generation alkylating chemotherapeutic agent related to imidazolotetrazines [3, 6, 7, 10]. TMZ is accepted in the treatment of glioblastoma multiforme and other nervous system tumors and also in advanced stages of malignant neuroendocrine neoplasia, melanoma, and colorectal carcinoma. It is an optimal cytostatic medicine for treating slowly growing pituitary tumors [6, 7]. It should be emphasized that TMZ has several unique properties, such as 100% oral bioavailability, an exceptional ability to rapid crossing the blood-brain barrier, and the absence of cell cycle specificity [5–11].

In TMZ treatment responders, pituitary tumors show shrinkage, softening, and friability, which facilitates their resection at reneurosurgery [9, 11]. Three patterns of radiographic changes are seen in MRI: tumour necrosis and hemorrhage, cystic change, and shrinkage [6]. In pathologic studies, post-TMZ tumours had better differentiation and a lower Ki-67 index, involved larger cells, and showed reduced mitotic activity. Intratumoral necrosis, hemorrhage, accumulation of connective tissue, focal fibrosis, inflammatory infiltration, and neuronal transformation were also observed. The changes mentioned above suggest that the drug had an antitumour effect and promoted cell differentiation and slower neoplastic growth [9, 11].

The optimal dose regimen as well as the schedule and period of treatment of aggressive pituitary adenoma have still not been strictly defined [8, 9]. The standard, original, and most frequently used therapeutic dose of TMZ is 150–200 mg/m^2^ for five of every 28 days. The number of cycles of treatment reported in the literature ranged from 2 to 26 [9].

Temozolomide is generally well tolerated. Some often reported adverse effects include nausea, vomiting, fatigue, headache, and constipation. Myelosuppression, anemia, leucopenia, agranulocytosis, lymphopenia, and thrombocytopenia occur in few patients [6, 9, 10].

*The aim of the study* was to demonstrate the effectiveness of TMZ treatment of invasive corticotroph adenoma in a patient with NS and to discuss these results referring to the literature concerning TMZ therapy in atypical, aggressive corticotropinomas.

## 2. Case Report

The authors report the case of a 56-year-old woman who was diagnosed with Cushing's disease 6 years earlier. At the moment of diagnosis, she presented a clinical picture of serious hypercortisolism with dramatic signs and symptoms including fatigue, weight loss, visceral obesity (BMI-29.5 kg/m^2^), “buffalo hump,” “moon face,” plethora, thin skin, hirsutism, hemorrhagic diathesis, purplish skin striae, proximal limbs muscle atrophy, and weakness as well as severe hypokalemia, hypertension, and secondary diabetes with high (about 70 U/day) insulin demand. A loss of cortisol circadian rhythm, fivefold increased urinary excretion of cortisol, and a lack of cortisol suppression after low and high dexamethasone dose were observed (Table 1). Magnetic resonance imaging (MRI) revealed a pituitary borderline microadenoma sized 9 × 7 mm. In somatostatin receptors scintigraphy (HYNIC-Tektrotyd 99mTc), a higher accumulation of isotope was found only in the anterior pituitary, and suspicion of ectopic Cushing's syndrome was excluded.

After 2 months of ketoconazole therapy, she underwent the first selective transsphenoidal adenomectomy. A postoperative pathologic exploration performed using an electron microscope revealed a sparsely granulated corticotroph tumour with an MIB-1 index of about 40%. After the neurosurgery, the patient experienced severe secondary hypoadrenalism (morning plasma cortisol level, 0.68 *μ*g/dL; ACTH <10 pg/mL), and she needed hydrocortisone replacement treatment for 22 months. The patient's general appearance and feeling improved and her blood pressure, serum potassium level, and glucose tolerance became normal.

However, after a 27-month period of remission, the patient developed recurrent hypercortisolism due to a regrowth of the tumour. Thirty-one months after the first neurosurgical intervention, the patient underwent a second, nontotal transsphenoidal reoperation, followed by stereotactic radiotherapy (system Brain Lab, one dose 20 Gy).

Because of a rapid regrowth of the pituitary tumour and the increase of hypercortisolism, the patient was then treated with mitotane (6 g/day) and ketoconazole (1200 mg/day) simultaneously for 3 months in order to prepare her to next surgery, but this treatment was ineffective. The third transsphenoidal neurosurgery was performed nine months after the second operation.

This time only a short and transient decline of cortisol to 1.7 *μ*g/dL was observed.

Afterwards, because of a continuing progression of the tumour and increasing hypercortisolism as well as medical treatment failures and a lack of further options of treating the patient using neurosurgery, a total bilateral adrenalectomy was performed endoscopically as a lifesaving procedure. Postoperative pathologic investigations revealed nodular hyperplasia of both adrenal cortexes.

Subsequently, seven months after adrenalectomy, the patient developed Nelson's syndrome with intense skin hyperpigmentation and aggressive pituitary tumour progression together with optic chiasm compression and penetration to the right cavernous sinus. Four months later, the fourth transsphenoidal adenomectomy was conducted; however, there was a rapid recurrence of the tumour with an extended expansion to the right cavernous sinus causing ophthalmoplegia and with penetration to the sphenoid sinus and suprasellar region and the compression of the optic chiasm causing the blindness of the right eye. Five months later, she underwent the fifth neurosurgery intervention, a right frontotemporal craniotomy with a subtotal suprasellar adenomectomy, which was followed by the withdrawal of ophthalmoplegia.

**Figure 1:**
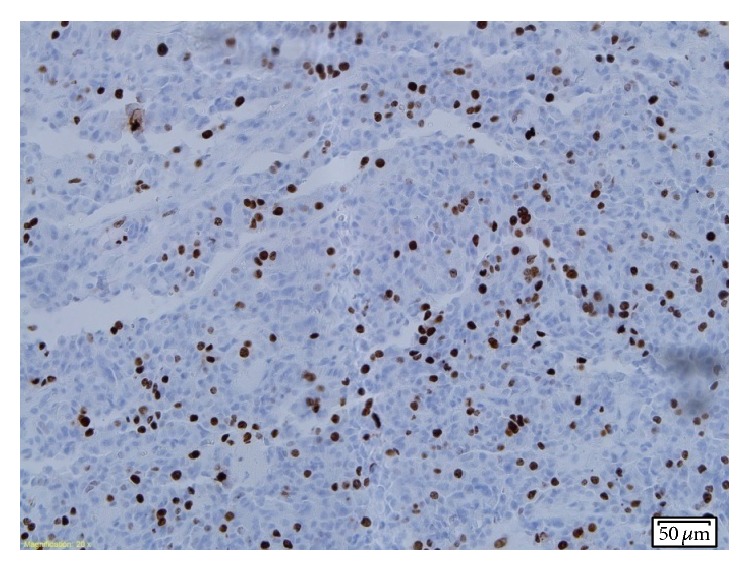
Nuclear staining for Ki-67 antigen (clone MIB1, Dako): MIB-1 proliferative index is greater than 5%.

**Figure 2:**
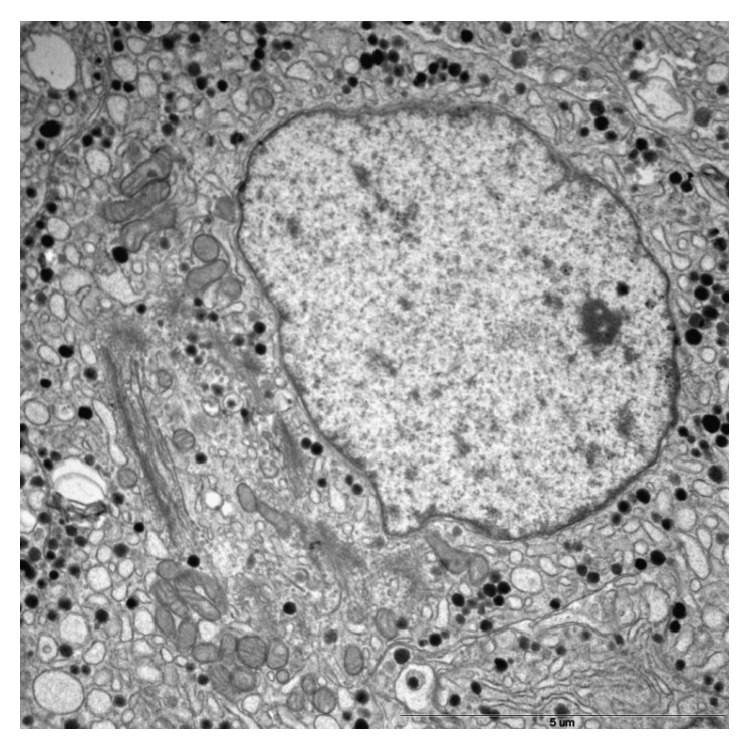
Ultrastructural features of sparsely granulated corticotroph adenoma (original magnification, 9700x). The cell has well developed Golgi complexes, dilated endoplasmic reticulum, and sparse, variable in shape and electron density secretory granules that measure 200 to 250 nm in diameter. Perinuclear bundles of cytokeratin filaments are absent.

**Figure 3:**
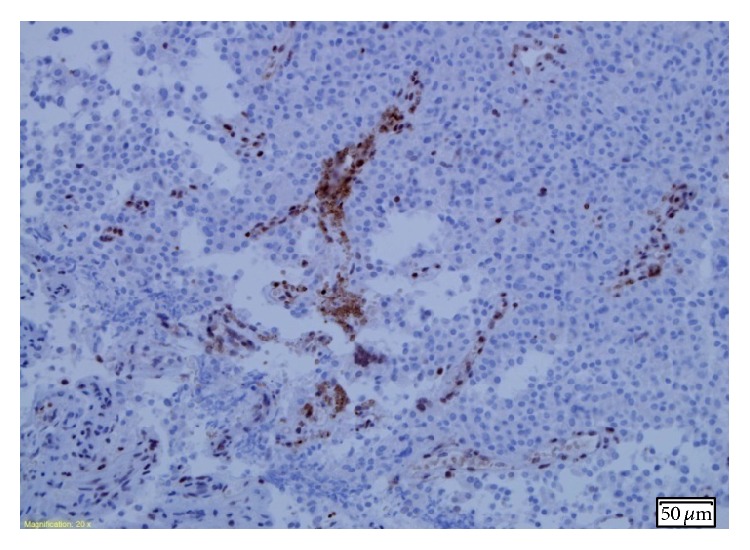
Positive MGMT reaction was observed in endothelial cells and only in single tumor cells. Activity of MGMT in tumor cells was assessed as very low.

A consecutive pathologic electron microscopy showed an atypical sparsely granulated corticotroph tumour, with an MIB-1 index >5% (Figures 1 and 2), very low activity of O^6^-methylguanine DNA methyltransferase (MGMT) in tumour cells (Figure 3), and positive receptor SSRT 2A (weak cytoplasmic) reaction. In order to help bridge the time gap until gamma-knife qualification, another desperate attempt of medical treatment was undertaken. Considering the fact that current literature proves the expression of somatostatin and dopamine receptors in corticotroph adenoma tissues [2, 18, 19] and taking into account the result of the somatostatin receptor scintigraphy and postoperative tumour receptor SSRT 2A examination, the patient was administered 2 injections of long-acting release somatostatin analog (lanreotide 120 mg) at a 4-week interval and cabergoline (2 mg/week) for 8 weeks. No positive effect was observed.

What made the situation even more difficult was the fact that, three months later, the patient was not qualified for gamma-knife therapy as the size of the pituitary tumour was too large.

Based on reports from the literature which showed a positive response in similar cases [2, 3, 5–11, 14–16], the second-line treatment with temozolomide was undertaken.

The patient received the most frequently recommended dose of TMZ = 150 mg/m^2^ of body surface area daily (total dose = 250 mg/day), administered orally for 5 days every 28 days.

Before each cycle of treatment, the clinical status of the patient was assessed as well as measuring the level of ACTH. MRI examinations were performed after the third and the sixth cycles. Tumour volume was measured based on the MRI picture using the computer program OsiriX. Treatment efficacy was evaluated after every three cycles.

**Figure 4:**
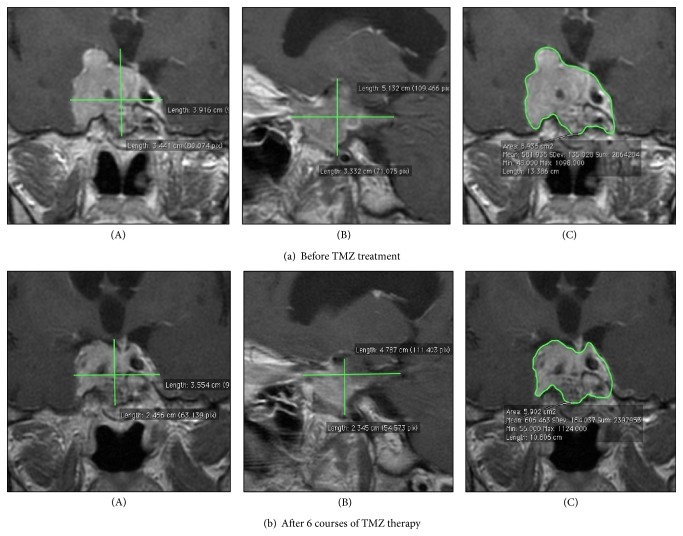
The assessment of tumour size (A, B) based on the MRI picture. The tumour volume (C) was measured using the computer program OsiriX. Frontal (A, C) and sagittal (B) scans.

When the TMZ treatment started, the patient complained about recurrent headaches and blindness of the right eye (earlier, after the neurosurgery, she had light sensitivity and could see the contours of objects). MRI scans performed before TMZ therapy are presented in Figure 4. Initial maximal tumour dimensions were 3.441 × 3.916 cm in frontal (Figure 4(a): (A)) and 5.132 × 3.332 cm (Figure 4(a): (B)) in sagittal images and its volume was 16.2 mL (Figure 4(a): (C)).

TMZ was well tolerated: only nausea and vomiting were observed. After the first cycle of therapy, light sensitivity in the patient's right eye recurred. Simultaneously, the level of ACTH in the serum was reduced by half of its initial concentration.

After three cycles, the patient reported further improvement of vision and an MRI examination revealed a reduction of tumour volume of about 3.2 mL (19.8%). The ACTH concentration in the serum was also lower.

After six cycles, the improvement of vision and general condition of the patient were obvious and well-marked. MRI scans showed a further reduction of tumour size (3.554 × 2.466 cm) (Figure 4(b): (A)) and 4.787 × 2.345 cm (Figure 4(b): (B)) in frontal and sagittal images, respectively, and a reduction of volume of about 1,21 mL (Figure 4(b): (C)). A total reduction of tumour volume after six cycles received 4.4 mL (27.3%) and the ACTH concentration decreased to 1/4 of its initial value. The results of TMZ treatment are shown in Table 2.

All in all, till September 2012, the patient received 9 cycles of TMZ without progression of the disease and with a stable volume of the tumour.

Unfortunately, after TMZ withdrawal and a 6-month period of remission, the tumour progression causing ophthalmoplegia and the blindness of the right eye relapsed. Then bevacizumab (anti-VEGF antibody) was introduced. Transient clinical stabilization of the disease has been observed. The patient died after another neurosurgery intervention, propter postsurgical complications.

## 3. Discussion

The patient described in this article is the first person in Poland treated with temozolomide for invasive pituitary tumour. At the same time, she is the first patient in our country treated with TMZ due to an atypical corticotroph pituitary adenoma and the first person in Poland to whom TMZ was administered to treat an adenoma occurring in Nelson's syndrome.

Corticotropinomas are more invasive than other types of pituitary tumours [2, 8]. In a study by Zada et al. [20], patients with atypical adenomas accounted for 15% of the whole group with pituitary tumours and 27% of them were corticotropinomas. Since 2006, a total of over 70 patients with pituitary tumours have been reported to have been treated with TMZ in isolated clinical cases or in retrospective studies in the English literature [6, 21, 22]. These cases included as many as 24 (35%) patients with corticotropinomas. Sixteen (66%) of them had atypical pituitary adenomas [6, 21, 22]. Only six patients with NS seem to have been treated with TMZ so far [10, 11, 14–17].

Intense symptoms of hypercortisolemia, a reduction (not an increase, which is usually the case) in body mass, severe hypertension, and unstable diabetes as well as severe hypokalemia which were present in our patient from the onset of the disease suggested aggressive adenoma. We found a lack of suppression of cortisol in both low- and high-dose dexamethasone tests, which, according to Barber et al. [2], could indicate an invasive nature of the pituitary tumour and anticipate the subsequent development of NS.

Another feature which suggested an exceptional invasiveness of the pituitary tumour in our patient was its relatively large size at the moment of diagnosis. Our suspicions based on the clinical picture were confirmed in the postsurgery pathological analysis: it demonstrated an atypical, sparsely granulated corticotroph adenoma with a very high MIB-1 labeling index. It was previously documented that a nuclear antigen Ki-67 of over 3% (identified with MIB-1 antibody) exhibits a significant association with the invasiveness of pituitary tumours [7, 20, 21, 23]. The sparsely granulated form of ACTH pituitary adenoma is also more invasive than its classical densely granulated type [23, 24].

Postoperative secondary adrenal insufficiency which required the substitution treatment for several months seemed to confirm the effectiveness of the neurosurgical procedure [25, 26].

When the disease relapsed, the tumour became significantly more aggressive. Due to a faster progression of the adenoma, accompanied by a massive invasion of the surrounding structures, the patient underwent two more neurosurgical procedures. Another postoperative histopathological analysis, carried out after NS had been diagnosed, additionally confirmed sparsely granulated atypical corticotroph adenoma with increased markers of proliferation.

Based on the published data, TMZ is recommended in the treatment of aggressive ACTH-secreting tumours, including those in NS resistant to other therapeutic options [1–16, 21, 22]. Ortiz et al. [6] reported that 60% of pituitary adenomas and 69% of pituitary carcinomas responded favorably to TMZ treatment. In a study by McCormack et al. [27] ACTH secreting tumours belonged to the higher response group, with a response rate of 60%.

Raverot et al. [21] observed a decrease in ACTH secretion in 67% and reduction of tumour volume in 56% of patients with atypical corticotroph adenomas after 9.1 ± 4.7 cycles of TMZ. In patients responding to TMZ, tumour shrinkage and a reduction of hormonal secretion were observed within few weeks [6–8]. The response to treatment may be dramatic and sustained; however, a lack of response after 3 cycles suggests resistance to the drug [20]. Moreover, the initial response to TMZ is not always associated with long-term tumour control and tumour relapse during TMZ therapy was also described [21, 27].

The small number of patients treated so far, the application of different treatment modalities, and the use of heterogenic criteria to estimate treatment effects make it difficult to analyze and compare the results of TMZ therapy with multiple independent case reports.

The response of pituitary tumours to cytostatic treatment has not been defined in a uniform way. Raverot et al. [21] defined tumour response as a 20% decrease in maximum tumour size and hormone response as a 50% decrease in hormonal secretion. Losa et al. [11], on the other hand, described a positive response as a reduction in tumour size of at least 50%, the tumour being measured in the greatest length and maximum width of all lesions, and a normalization of hormone hypersecretion, while the disease was considered stable when a <50% reduction in tumour size and hormone hypersecretion were found.

In the majority of published cases, the reduction of tumour size was assessed inaccurately as the assessment was based only on the measurement of the maximum dimensions of the tumour. The authors of the present report, similarly to Bush et al. [28] and Curtò et al. [29], have applied computer measurement of volume which made it possible to precisely determine tumour volume both before and after TMZ therapy and allowed for an accurate and comparable estimation of the treatment results. Following Raverot et al. [21], we used a 20% tumour volume reduction as a positive criterion of tumour treatment response in MRI, which was estimated using the computer program.

Temozolomide has different degrees of efficacy in treating aggressive pituitary tumours. It can have a varying impact on tumour size: it can cause a rapid and considerable reduction, a moderate reduction with subsequent stabilization of tumour size, or only an arrest of tumour growth [5, 7–9, 11, 28–30]. Dillard et al. [8] reported a case of aggressive corticotroph adenoma with a 60% decrease in tumour size and a regression of cranial nerve palsy after 10 weeks of TMZ therapy. Bush et al. [28] observed a regression of tumour volume of more than 80% with rapid clinical improvement within the first four cycles and steady decrease of ACTH levels in a patient with corticotroph pituitary tumour and Cushing's disease. Curtò et al. [29] reported a dramatic (over 90%) tumour size regression of ACTH-secreting carcinoma after four cycles of TMZ.

Raverot et al. [9] described four patients with ACTH-secreting pituitary tumours treated with TMZ. They observed significant tumoural, hormonal, and clinical responses in two patients after only four cycles of TMZ. In the other two cases, despite 7 to 14 cycles of TMZ, no tumour or hormone response was noted, even when it was accompanied by carmustine or carboplatin.

The findings presented above motivated us to administer TMZ to our patient in the most commonly used, conventional schedule and dose [3, 6–8, 11]. Similarly, as in the cases described above [6, 8, 9, 11, 14, 28], the patient responded to treatment quickly. As soon as after the first cycle, ACTH levels were found to have decreased by half. A tumour response (a reduction of about 20% in tumour size) was observed after 3 cycles along with a continued decrease in ACTH level. These changes were accompanied by considerable improvement in the clinical state of the patient, including a release of the compression on the optic chiasm. The next 3 cycles brought about a further though less considerable (only about 10%) reduction in tumour size and a decrease in ACTH levels as well as further improvement of the general state of the patient and of the vision in her right eye. Similarly, as in other cases [6, 8, 9, 11, 14, 28], the patient tolerated the treatment well, with only a mild increase in the side effects.

Due to the invasive nature of the adenoma, in Nelson's syndrome, the reduction in tumour size is more significant from a clinical point of view than the decrease in ACTH levels. In a patient with NS in the study by Moyes et al. [14], TMZ treatment resulted in significant clinical and hormonal improvement and shrinkage of the tumour after only 4 cycles of treatment. Takeshita et al. [15] reported a case of Crook's cell carcinoma and a rare form of Nelson's syndrome induced by the adrenolytic effect of mitotane. After 6 cycles of TMZ, the authors observed clinical improvement with a significant decrease in ACTH and cortisol levels and only marked shrinkage of the tumour. The patient described by Losa et al. [11] had only a temporary slight reduction of the inferior part of tumour and a transient hormone response followed by a stabilization of tumour growth. Mohammed et al. [16] achieved a good response in their Nelson's patient after 12 courses of TMZ, but the disease relapsed once therapy was discontinued. Only one patient from the study by Raverot et al. [9], who had had a bilateral adrenalectomy but without NS, did not respond to TMZ therapy. Annamalai et al. [30] reported a case of a patient with silent corticotroph adenoma which subsequently passed to overt Cushing's syndrome. TMZ therapy applied before bilateral adrenalectomy resulted in a marked clinical, biochemical, and radiological response. However, immediately after the adrenalectomy, despite reintroduction of further eight cycles of TMZ, positive effects of therapy were no longer observed.

In the majority of cases of atypical corticotropinomas and Nelson's syndrome reported so far, including that of our patient, temozolomide has been demonstrated to have a heterogenic effect.

Although the response rates were different in every case, TMZ prolonged survival, induced significant clinical improvement, and ameliorated the quality of life [7]. Raverot et al. [21] reported that temozolomide was a unique medication in terms of its ability to control atypical pituitary tumours in about 60% of patients, but, according to their latest observation of 24 patients with aggressive pituitary tumours (16 adenomas and 8 carcinomas), temozolomide response ratio was only about 40% [22].

Activity of O^6^-methylguanine DNA methyltransferase (MGMT) in the tumour cells in our patient was assessed as very low. Because the low activity of MGMT, defined as <5% nuclear MGMT staining [15], is generally regarded as a predictive marker of sensibility and a favorable clinical outcome in patients with temozolomide-treated aggressive pituitary adenomas [15, 22, 27, 28], we have postulated that the assessment of MGMT immunoexpression should be a standard component of a postsurgical specimen study in patients after a second adenomectomy because of suspecting the atypical adenoma. This information may be useful in the future to qualify patients for therapy with TMZ.

Other effective ways of treatment in invasive corticotropinomas can be temozolomide in combination with capecitabine [31, 32], bevacizumab [33, 34], and everolimus [17].

We agree with other clinicians [6–11, 14, 21, 29, 30] that temozolomide is at present the most efficacious, available, easy to use, and well tolerated medication of low toxicity that can be used as a last-line treatment for life-threatening pituitary tumours, which are refractory to standard treatment modalities.

## 4. Conclusion

Temozolomide should be considered and prescribed as an effective agent in the medical treatment of aggressive corticotroph adenomas and in Nelson's syndrome resistant to other treatment options.

## Figures and Tables

**Table 1:** Laboratory tests in patient at the moment of diagnosing Cushing's syndrome.

Diagnostic test	Normal range^*∗*^	Patient result
ACTH (pg/mL) 8.00 am	<46.0	81.0
Cortisol (*μ*g/dL) 8.00 am	4.3–22.4	54.45
Cortisol (*μ*g/dL) 6.00 pm	3.09–16.66	52.72
Urinary excretion of cortisol/24 h (*μ*g/dL)	32–243	825.6; 1275.5
Cortisol (*μ*g/dL) after 8 mg DXM suppression test	↓50%	54.45 → 41.2 [↓24%]

^*∗*^According to local laboratory 2006.

**Table 2:** The process of TMZ treatment of corticotroph pituitary adenoma in Nelson's syndrome in our patient.

Stage of treatment with TMZ	Clinical symptoms	ACTH (pg/mL) normal range^*∗*^ 7.2–63.3	MRI, volume (mL) of the tumor
Before treatment	Recurrent headaches and blindness of the right eye due to optic chiasm compression	23830	16.137

After the first cycle	Return of light sensitivity in right eye	12110	—

After three cycles	Further conspicuous improvement of vision in right eye. Improvement of general appearance and feeling and reduction of hypotension drugs and daily insulin doses	9215	12.935 Reduction in tumor volume of 19.8% (3.202 mL)

After six cycles	Further improvement of vision in right eye and general condition of the patient	5712	11.725 Further reduction in tumor volume of 9.3% (1.21 mL)

^*∗*^According to local laboratory 2012.
